# Early childhood education in Africa: Between overambitious global objectives, the need to reflect local interests, and educational choices

**DOI:** 10.1007/s11125-022-09608-7

**Published:** 2022-08-08

**Authors:** Abdeljalil Akkari

**Affiliations:** University of Geneva, Uni Mail, 40 Bd du Pont-d’Arve, 1211 Geneva 4, Switzerland

**Keywords:** Pre-primary education, Africa, International cooperation

## Abstract

The African continent represents the part of the world where early childhood care and education (ECCE) preceding primary schooling is the least developed. It is therefore essential to propose an analysis of the prospects for the development of early childhood education in the coming years. This article deals in the first instance with the international infatuation for pre-primary education based on both scientific evidence and also international agendas. It then explores the possibilities of putting forward a coherent African response to global demands concerning early childhood education and promoting the inclusion of local cultural content in designing pre-primary educational structures. Finally, the article suggests some principles that might guide international cooperation activities affecting the early childhood sector.

## The global infatuation for early education

The global infatuation for ECCE has not weakened over recent decades, as is illustrated by the latest global reports reviewing education. The arguments put forward in this discussion are numerous and deserve review and constant updating in relation to both the situation in the field and also the outcomes of scientific research.

First, let us examine the scientific evidence, which may be divided into two parts. The first concerns the development of the brain during the first years of life. The second deals with the benefits ascribed to investment in early childhood education. The importance of the brain’s development during an individual’s first years of life is now accepted. During the first months, children begin to construct their strength, postural stance, and coordination. They develop precise benchmarks for time and space that will be employed throughout life, as much for movement as for carrying out numerous tasks, particularly for learning in a school environment. It is during the first years of human development that the structure and the basic functioning of the brain take form. The period of development at the beginning of childhood (from conception to the age of 6 to 8 years) therefore has a major impact on the subsequent stages of human development (Mustard, 2010). The first years of life are vital for the subsequent psychoeducational stages that will be necessary later to compensate for development any missed opportninities by the very young child. The course of life during the first years therefore constitutes either a solid or fragile foundation on which all the subsequent development of the child, the adolescent, and the adult will be constructed (Shonkoff & Phillips, 2000).

A large number of long-term research studies in numerous national contexts, both in the global North and the South, have drawn attention to the rate of return on investments in early education (Heckman & Masterov, 2007). One of the most well-known of these studies is without doubt the *Perry Preschool Study*, carried out in Michigan between 1962 and 1967 among a disadvantaged population, a study that has been constantly updated ever since to follow the evolution of these children over time. It highlights the long-term positive outcomes of a quality education during early childhood (Schweinhart & Weikart, 1993).

Another significant argument for pre-primary development is its contribution to school readiness, the prevention of repetition, and the strengthening of school performance (Charles-McCoy et al., 2018; Currie, 2001).

Without contesting Africa’s interest in these scientific findings, two reservations should be expressed. Firstly, while the development of the brain needs educational stimulation, it also needs, and this should not be overlooked, access to health services, adequate food, and beneficial living conditions. The magnitude and seriousness of the health and nutritional situation of African children remains alarming. A high percentage suffer from anemia and malnutrition, which has a consequent impact on the child’s psychomotor development (Aynalem et al., 2022).

It is therefore important to pay equal attention to all of the factors contributing to cerebral development and not to overestimate the impact of one factor over another. On this point, we regret that some public authorities in Africa think their job is done and they have a clear conscience by cramming dozens of very young children into a pre-primary class under precarious conditions conducted by an ill-trained or untrained educator. They delude themselves that, in this way, they have invested in early childhood. We may also observe that studies focusing on the benefits of investing in early childhood have generally been carried out in the countries of the North. Their methods, theoretical bases, and conclusions should be adapted to the situations found in the countries of the South (Penn, 2010, 2011).

Next, we encounter the arguments connected with the global educational objectives promoted by international organizations such as UNESCO, UNICEF, or the World Bank. For example, Sustainable Development Goal 4 (SDG4) recommends that all countries of the world should, by 2030, provide one year of good quality, free pre-primary education to all children. Among the 17 objectives of sustainable development, Objective 4 is aimed at quality education for all children in the world. Target 4.1 of this objective deals specifically with early childhood education and states that between now and 2030 countries must ensure that all girls and all boys have access to developmental activities and early childhood care, as well as to quality preschool education prior to entering primary education. The minimum requirement is to offer *at least one year of free and compulsory quality pre-primary education provided by well-trained teachers* (UNESCO, 2015a)*.*

We have recently carried out an analysis of the situation in the African countries which are members of CONFEMEN (*Conférence des ministres de l'éducation des états et gouvernements de la Francophonie*—Conference of Ministers of Education of French-Speaking States and Governments) to estimate their chances of reaching this international objective by 2030. The result is damning. With the exception of Mauritius, which has a gross enrollment rate in pre-primary education approaching 100%, the distance separating the African countries of CONFEMEN from SDG 4.2 is very wide. Six countries have a rate below 10%: Burkina Faso, Chad, Côte d’Ivoire, the Democratic Republic of the Congo, Mali, and Niger. Five countries have a rate between 10% and 25%: Benin, Burundi, Guinea, Senegal, and Togo. Two countries are placed between 30% and 40%: Madagascar and Cameroon (Akkari, 2021). If we broaden our perspective, it can be seen that certain English-speaking and Portuguese-speaking countries, such as Cape Verde, Ghana, Kenya, or the Seychelles have made significant progress toward broadening access to pre-primary education.

Between now and 2030, there is a risk that these African countries and international cooperation will be obliged to undertake the politics of numbers by rapidly increasing the quantity of pre-primary classes and children without paying attention to the financial and human resources, nor to the planning necessary for quantitative and qualitative growth in this sector. It follows that we need tools available to measure the quality of curricula and to establish a genuine curriculum for pre-primary education.

Finally, we believe it is useful to emphasize that the evolution in the international terminology employed in the field of early education shows a tendency toward “overshooling is better in early childhood” to the detriment of other possible and perhaps more reasonable approaches. If we look only at the French terminology, at least four terms are possible when speaking of formal education during early childhood: (a) *éducation pour la petite enfance* (early childhood education), (b) *éducation préscolaire* (preschool education), (c) *enseignement préprimaire* (the term employed in SDG4, pre-primary education), and (d) *école maternelle* (kindergarten). The first term is not necessarily associated with scholarity and may refer to any institution concerned with children during their first years of life. These institutions may, furthermore, be managed by ministries other than that for education, for example the ministry for families, for social development, or for health. This may also include community centers for education, or other structures designed to take care of children from a very young age, including those involving families. The terms *préscolaire* (preschool) and *préprimaire* (pre-primary) indicate clearly a structure concerned with children before they enter primary schooling. The purpose of these structures is to prepare children for entering primary education without hindrance. They usually fall under the ministry of education. The term *école maternelle* (kindergarten) covers the necessary transition between the family or parental milieu and the primary school. The use of the term *préprimaire* (pre-primary) in the context of the International Agenda 2030 stresses that the international priority is to create a place that anticipates schooling and stimulates the preparation and readiness of children to enter the school.

Two dangers may accompany this over-scholarization of early childhood. Firstly, it does not necessarily encourage children’s cognitive or socioemotional development. Secondly, it requires considerable human and financial resources. To sum up, it appears that the terminological diversity associated with early childhood education is the sign that the debate is not settled between a developmentalist and an educational vision of this sector (Marinova et al., 2020).

It is important to emphasize that the terminological discussion also refers to important teaching and educational problems. Early childhood is an age group providing its own services for a holistic and ambitious development of future generations. Furthermore, there is no agreement on the priority objectives since there is a tension between the developmental objectives of the children (stimulation, socialization, awakening, preparation) and the objectives of early schooling or school readiness.

## Viewing global imperatives from the local level

We have indicated some of the discussions and contradictions surrounding the global craze for early childhood education. It is useful to base the discussion of early childhood education on local and national realities. On this subject, it seems to us indispensable to recall a historical fact that comparative education has not ceased to highlight for a long time. All education systems have been developed on the solid foundation of *primary education,* sometimes called, with good reason, fundamental or basic education. All the other levels of education—pre-primary, secondary, and even higher education—have been based on and depend upon this initial foundation. In other words, it is extremely difficult, if not impossible, to develop a viable pre-primary sector without a reliable primary sector of quality. Unfortunately, this is far from being the case everywhere in Africa. Obviously, it is possible to accelerate the development process for pre-primary education, but this process will remain fragile as long as primary education has not been consolidated.

Globally, access to educational services for early childhood has doubled over recent decades, passing from a general average for all the countries of the world of approximately 30% of children in 1986 to an average of slightly over 60% in 2019 (UNESCO, 2021a). However, this increase appears to have taken place mostly in South Asian, East Asian, and Pacific countries, as well as Latin American and Caribbean countries. Middle Eastern and North African countries, as well as those in sub-Saharan Africa, exhibit on average percentages lower than that for other developing regions, which are limited on average to a gross rate of about 30% for pre-primary education. In brief, in 2019 the level of preschool development for African countries was situated at about the same level as the rest of the world was in 1986. It is therefore not reasonable to consider that they would reach the SDG4.2 target in 2030, less than eight years from now.

Overall, if the objective is to reach the targets of the 2030 Agenda, it would be sufficient to situate pre-primary education in Africa in the local context. Such a scenario requires several steps and preliminary measures to be carried out at the national level.

In the first place, it is essential that each African country arms itself with a national policy for children incorporating a holistic vision of the childhood sector (law, protection, health, food, education, etc.). (Depending on the country, there are other options than a specifically national policy for early childhood in a holistic manner. Indeed, it is possible to imagine a strong political connection between the government sectors that are concerned by early childhood: health, education, social policy. An integration of early childhood into national development plans is another path to explore) The question of equity is also a key element in any policies on childhood. We know that unequal opportunities are reflected in social, ethnic, and regional disparities. Public authorities should follow the principle of paying more attention to less-favored regions and children. A recent study by UNESCO (2021b) has drawn attention to the slowness of adopting legal frameworks for pre-primary education. In two-thirds of the world’s countries, there is no national, legal measure ensuring free and compulsory pre-primary education.

Secondly, it is essential to reflect upon the diversity of educational provision for early childhood. Indeed, this variety is reflected in administrative structure, with at least three major sectors in Africa: the private sector (It is by far the most important in terms of enrollment), the public sector, and the communities (including the Koranic school, very common in countries with a majority of Muslims). The first of these is at present the principal provider of education for children in Africa before entering primary school. Recent figures suggest that 55% of all children enrolled in pre-primary education in low-income countries are catered to by private providers (UNESCO, 2015b).

The public and communitarian sectors require national public financing and the support of the international community. Over recent years, the financing of the GPE (Global Partnership for Education) has triggered a true surge in this sector, particularly in those contexts affected by vulnerability and urgence (Silva & Oliveira, 2021).

For the early childhood sectors, in the public as well as the private and communitarian domains, the roles of the state and the ministry of education remain indispensable in every country for the control and management of that sector: infrastructure, teacher training, establishment of the curricula, and objectives. Table 1 sums up the advantages and limitations for the three principal sectors.

**Table 1:** Advantages and limitations for the three principal sectors of early childhood education

Sector	Advantages	Limitations
Public	Allows the state to trial major educational innovations Can provide means for greater equity (rural and disadvantaged children) Provides articulation with public primary schooling Could connect immediately to the primary school	Carries significant cost for public finances Risks bureaucracy and centralization of the sector May ignore regional and local characteristics
Private	Relieves the state budget through financing primary education by families Offers a place to try out innovations	Is only found in regions where there is a demand (parents willing to pay) May escape from state control and regulation
Communitarian	Provides proximity to parents’ needs Requires less investment Carries less risk of overschooling	May accumulate a disadvantaged clientele May escape from state control and regulation Places a huge burden on fragile communities

*Source*: The author

Our recent observations in this field over the last few years lead us to foresee diversification in the provision of preschool education in Africa, as well as avoidance of rapid and frequent changes on this matter (legislation, rules, standards, etc.). Furthermore, the three major sectors we have highlighted above are made up of a multitude of subsectors, which must also be examined and analyzed in the near future.

Thus, the public sector could consist of three fairly different forms of pre-primary education:

- A pre-primary school in a separate building from the primary school and managed by the ministry of education;
- A kindergarten class (sometimes called a preparatory class) located in the same building as the primary school and managed by the ministry of education;
- A pre-primary school (sometimes called a kindergarten) managed by a local authority (village/town/region).

The private sector, basically urban, may consist of:

- A private pre-primary school for the affluent similar to those found in the North. In Africa, pre-primary schools for foreign missions correspond to this category (The schools of foreign missions—for example, French schools in Africa—represent a typical example of the scrambling of frontiers between public and private educational sectors. They are *public* for French citizens and *private* for the local citizens);
- A private pre-primary school for the middle class providing an indispensable childcare service for women increasingly involved in professional activities;
- A private pre-primary school for disadvantaged communities, sometimes not recognized by the state, and often escaping the regulatory authority of the educational administration.

The communitarian sector may include:

- Communitarian care and developmental centers for preschool-age children financed by the state or more often by international cooperation; these structures depend strongly on the commitment of local communities;
- Religious communitarian schools, including Koranic schools found throughout West and East Africa;
- Pre-primary schools financed and managed by national or international nongovernmental organizations.

Given the fragmentation of this sector into numerous different structures, an enormous effort is required for a statistical understanding of the pre-primary supply operating in the majority of African countries. On this subject, the study by Fuentes (2021) draws attention to the fragmentation of this sector in Senegal but also to the heterogeneity in the quality of present structures.

It would appear counterproductive to confront the public and private spheres in Africa, but one can emphasize the necessity of considering early childhood education as a public good fundamentally financed by the public. In other words, it is a service of benefit for the whole society, made available to all those who could benefit from it and provided by public, private, and communitarian institutions (Lawrence & Sharrock, 2021).

## A culturally relevant strategy for early childhood education

In a recent work (Akkari & Fuentes, 2021), we called for a reappraisal of indigenous knowledge in education and teaching so as to respond better to the challenges of the twenty-first century. It is probable that pre-primary is the educational sector which has the greatest need to be based on local pedagogy, traditions and cultures. As Pence and Marfo (2008) have rightly pointed out, a major challenge for early childhood education consists of African education specialists’ passive and uncritical consumption of knowledge and traditions arising from cultural contexts that have completely different values and hypotheses about childhood and human development.

Our various action-research projects in the field of early childhood education have also encouraged us to consider that certain themes should be the subject of a more profound discussion so that early childhood educational policies should evolve in the right direction (Awopegba et al., 2013). Firstly, we should abandon cognitive imperialism and epistemological injustices, which have arisen above all as the outcome of a move to belittle colonized cultures, where the colonized people have been treated like children, if not barbarians (Bhargava, 2013). It is therefore essential to accept that the principal educational resources for pre-primary education should be drawn from the local culture. It is indispensable to explore the values, beliefs, and ethnotheories associated with the conception of childhood. To bring ideas in from elsewhere drawn from scientific evidence is, of course, also indispensable, but these ideas must be grafted on to the local cultural stock. On this subject, we should not underestimate the resistance of preschool personnel in Africa to look within their own cultural heritage. In a long-term action-research project we have been conducting in Madagascar over several years, we were surprised by the lack of local children’s games for preschool children in the pre-primary classes. When we asked the Malagasy teachers if there were any traditional games suitable for very young children, the answer consistently expressed by them was that there were none. However, all we needed was to go out into the playground or to wander through the residential neighborhoods to discover a multitude of games played by Malagasy children of all ages in complete liberty.

Indeed, the educators replied by thinking that the only educational games *worthy of consideration* were those employed in Western kindergartens (puzzles, memory games, dice games, Lego, pop-up books etc.) to develop memory through images and sensorimotor skills. By inviting the teachers and the local researchers to work together to find traditional Malagasy games potentially useful for preschool education, we identified almost 100 games played by the country’s children, of which we selected about 30 that were included in a guidebook intended for Malagasy pre-primary educators (Akkari & Rakotozafy, 2014). African cultures are a largely unexplored source of innovation for preschool, and the indigenous approach could strengthen the development of a culturally appropriate education for early childhood: “The Indigenous Early Childhood Care and Education framework is a strength-based approach that acknowledges and seeks to incorporate the knowledge, skills, values, and timeless wisdom of early child care and education that originated in Africa and are still usefully relevant for rapidly globalizing requirements for early childhood development” (Awopegba et al., 2013, p. 37).

Next, we estimate that the choice of a language that the children can understand (maternal or first language) is an advantage supported by both research (Cummins, 1986; Skutnabb-Kangas, 2001) and international organizations (UNESCO, 2015a; World Bank, 2021). Children learn better and are more likely to pursue their subsequent studies when they have begun their schooling in a language that they use and understand. However, according to international estimates, 37% of children in low- and medium-income countries are obliged to learn in a language other than their mother tongue, which places them at a considerable disadvantage throughout their schooling and limits their ability to learn. The children most affected by these questionable policies and choices often come from disadvantaged societies. Socioeconomically, they are located in the lowest 40% of households and live in rural or remote regions. Furthermore, they do not have access in their family context to the resources that could overcome the impact of unsuitable learning policies. The result of all these factors is higher dropout and repetition rates, as well as a deficiency of learning and a diminished general level of instruction (World Bank, 2021).

The new interesting approach adopted by the World Bank (2021) toward the teaching language is based on five principles:

- To ensure teaching in the pupils’ first language until at least the end of the sixth year of primary education.
- To use the pupils’ first language for teaching subjects other than reading and writing.
- When the children have to learn a second language during primary education, to proceed with the teaching of this language as if it were a foreign language by first stressing oral skills.
- To continue with teaching in the first language, even when the second language has become the principal teaching language.
- To plan, develop, adapt, and systematically improve the introduction of policies concerning the teaching language, taking into consideration the national context and the educational objectives.

This approach, undertaken by the organization with the most influence on education in the countries of the South, is relevant but cannot avoid meeting foreseeable difficulties in the field. The results of a study carried out in Zimbabwe, for example, showed that 86% of parents preferred English to be used as the teaching language in early childhood education. These parents considered it useful to introduce English as early as possible so their children would acquire this language, which would influence the remainder of their schooling due to its intensive use in the primary school (Ephias et al., 2015).

One way to implement the first principle of the World Bank’s new approach (“To ensure teaching in the pupils’ first language until at least the end of the sixth year of primary education”) is to include in national educational policies the obligation that all primary schools (including private schools serving local elites) should teach in the pupil’s mother tongue or in a widespread and active national language. (If we restrict ourselves to two West African countries, it is possible to choose Wolof in Senegal and Hausa in Niger as teaching languages for the first years of schooling.)

It is also important to introduce new policies for training early childhood educators and to launch the production of teaching resources in the mother tongue. As for the educators, it is essential they adopt the strategy of alternating theory and practice so that they may, in particular, accumulate teaching resources based on the local culture. These will play a primary role in preparing children to acquire the written culture.

Furthermore, the curricula and the pre-service training of pre-primary education educators must receive greater attention than at present. The experience of Reggio Emilia in Italy is most instructive (Edwards & Gandini, 2018). The Reggio approach is based on fundamental principles describing a certain number of professional themes and attitudes. The whole procedure forms a coherent teaching system making the children independent and happy to discover, explore, and learn. The educators share this optimism and confidence in the children’s learning capacity, recognizing that children possess extraordinary resources and potential. They are capable of constructing their thoughts, questions, and attempts to find answers by themselves. The children observe objects and situations and reconstruct them. They express themselves through different languages, thereby generating new meaning, and seek to master the way those around them express themselves (Dubois, 2015). With the exception of South Africa, the African continent seems to be the region of the world that has not yet tried out the Reggio Emilia approach (Foerch & Iuspa, 2016). Nevertheless, this approach has vast potential in the continent, particularly in understanding the hundreds of languages of the Malaguzzi children (Mphahlele, 2019). Setting the work in context obviously remains to be accomplished to adapt this approach to the African situation.

Marinova et al. (2020) identify two educational approaches in early childhood. One of them is the developmental approach to preschool education, which takes the form of educational practices based on assisting the child in their developmental activities and interests, and which builds upon the young child’s natural aptitudes to learn, particularly through games and experimentation. The other is the schooling approach, which follows various behavioral and cognitivist models. It takes the form of educational practices based on the transfer of knowledge, such as direct and explicit teaching, rote learning, and exercises with paper and pencil. The study by Marinova et al. highlights that the preference of teachers for learning the written language is more in favor of the schooling approach. We believe it is important not to place too much emphasis on the schooling approach during preschool education in Africa, which finds its justification in the need to prepare children as quickly as possible for a teaching language with which they are not always familiar.

Entry into primary education represents a first step in children’s school careers and the first in a long series of transitions for them. Indeed, advancing to the school represents a critical stage in the life of any family. It is the first opportunity for the children and their families to experience the activities connected with this transition to both the school and the future educational environment, and to exchange information about each child (CTREQ, 2018).

Furthermore, the theme of an appropriate infrastructure for pre-primary education entirely sums up all the challenges faced by this sector. In a study on developing early childhood education in Laos, we were able to observe the excellent premises proposed by Japanese cooperation in the rural areas surrounding the capital Vientiane. In Africa, unfortunately, our conclusions in the field are irrefutable. Precarious classes in straw huts and sometimes under a plastic tarpaulin, as we have recently seen in Niamey, were supposed to shelter children whose schools had been flooded. There was an absence or malfunction of toilets and limited access to drinking water. If it were impossible to conduct these pre-primary classes in a place suitable for the development of the children’s education, it would have been better to leave the children at home to grow up in their family and community environment. The infrastructure should identify a number of conditions vital for the quality of preschool education in Africa: the pupil/teacher ratio and the space available for each child in the classroom. The first number should under no circumstances exceed 30 pupils per educator, and the second should guarantee 1.5 meters per child and never less than 1 meter. Furthermore, the environment surrounding the class—pleasant, safe, and healthy—is also vital for a pre-primary education of quality. We have noted that during the rainy season pre-primary classes are not accessible to pre-primary children and the toilets are often out of commission—if they exist at all.

On the subject of infrastructure, it is also necessary not to overlook the children living in numerous conflict zones in Africa. The presence of conflicts has been associated with a drop of 5.9% in the probability of a child being on the path toward good development from the first year of exposure. The lack of access to early childhood education has a serious impact on socio-emotional development (Goto et al., 2021).

Finally, we should not limit our concern for early childhood education to one age group (usually, that preceding entry to primary school; in other words, 4 to 5 years) or to a typical space (most often the formal education institution). It is useful also to pay attention to the lack of learning and stimulating opportunities for the children in the family environment. A study by Charles-McCoy et al. (2018) drew attention to the low percentage of African children who enjoy suitable stimulation in the home and an opportunity to attend an educational structure for early childhood comparable to that in other regions of the world, as shown in Table 2.

**Table 2:** Learning opportunities by region and by country income

	Number of countries	% with high stimulation	% in ECCE	% with low stimulation & no ECCE	% with high stimulation & ECCE
*Region (World Bank)*					
East Asia & Pacific	5	76.1	48.3	15.3	39.7
Europe & Central Asia	14	88.6	37.7	9.3	35.5
Latin America & Caribbean	12	86.2	61.7	8.2	55.9
Middle East & North Africa	7	63.1	22.1	28.4	16.1
South Asia	4	68.6	18.9	27.7	15.2
Sub-Saharan Africa	21	54.0	17.9	37.1	11.1
Low income	16	53.9	14.0	38.6	9.2
Low/middle income	23	70.8	29.0	22.6	22.8
High/middle Income	19	83.4	47.1	11.6	42.1
High income	5	87.5	65.8	7.4	60.7
Total/average	63	71.9	33.6	22.1	28.2

*Source*: Charles-McCoy et al. (2018)

## Principles for action by international cooperation in the early childhood sector

At present, international cooperation plays a key role in the development and direction of early childhood education in Africa. It is therefore essential to suggest some principles which may guide its activities. At present, numerous African countries have turned to international cooperation to support them in planning, financing, structuring, and developing early childhood education. Nevertheless, Acharibasam & McVittie (2020) have clearly identified the tensions between traditional African methods for raising children and the present model for early childhood education in the global South. These divisions have come to the fore particularly because the theories supporting international programs do not correspond to the local culture. On what principles should international cooperation rely to lessen these tensions?

Firstly, the financing of early childhood education should gradually be assumed by the national budget. International aid can trigger an initial decisive start but, in the long term, domestic financing of this sector is necessary. Due to the lack of preschool education available in rural areas, public financing should concentrate on these regions while leaving the private sector to propose diversified offers in urban areas. This approach also means helping the poorest families living in urban areas deal with the financial burden of private preschool education.

Secondly, it is necessary to carry out a systematic analysis of this sector preliminary to its development. Too frequently, this diagnostic phase is rushed through because one is trapped in *the total belief in the unlimited benefits of early preschool education.* This analysis should examine particularly the capacity of primary schools to support the expansion of pre-primary education and the availability of qualified staff to supervise the children in an appropriate manner.

Thirdly, it is important to encourage innovations that can be scaled to correspond to local and national resources. Too often, a few dozen classrooms are constructed with the important participation of international cooperation, while overlooking the conditions for their long-term maintenance. It is therefore vital to see what happens to preschool classes provided by the GPE over recent years.

Fourth, while the diversity of educational provision for early childhood education is important, it is equally important that this provision should be based on local languages and culture (Serpell & Nsamenang, 2017). There should be a balance between national and international expertise. African universities should be more involved in reflection about this sector.

Fifth, minimum quality standards for the infrastructure must be set by the state. The pre-primary curriculum, as well as the pre-service and in-service training plans for the staff looking after the children, must be drawn up, approved, and actually applied in the field. The recruitment and training of educators must represent the cornerstone of quality standards for pre-primary education. The supply of integrated educational services for early childhood demands an inter-sectoral approach and a multitude of staff directed by the state. There should also be planning, coordination, and collaboration between ministries, civil organizations, businesses, and nongovernmental organizations.

Lastly, it seems to us essential to carry out borrowings, transfers, and adaptations of good practices for early childhood education *from the South to the South*. Africa may find more inspiration in early childhood education programs in South Asia or Latin America rather than in Europe or North America. The example of the nongovernmental organization BRAC International, which has been able to bring its valuable experience in this sector to Bangladesh and in Africa, merits our entire attention (Cronin, 2008; Smillie, 2009).

## Conclusion

We believe that the discussion on education during early childhood must remain ongoing and even intensify over the coming years (UNESCO, 2015c). Far from the assurances of the dominant viewpoints asserting that the miraculous solution to improve the quality of basic education in Africa is preschool education, we have shown in this article the need to moderate our enthusiasm and deal with this matter in depth. The slogan should not be “we must develop pre-primary education quickly” but “we must develop a sustainable pre-primary education in the context of an overall national vision of childhood, of basic education based on local resources (including cultural ones), guided by national and international research”. Pre-primary education has the particular responsibility to encourage a reliable transition by stressing the continuity and the quality of contacts between those involved (CTREQ, 2018), particularly in the situation following the Covid-19 pandemic (Nugroho et al., 2021).

It is therefore essential to develop partnerships and action-research allowing us to compare the impact of different methods on the development of children and their integration into the school. The contribution of international organizations is fundamental on condition that they listen to what is going on in the field and to those involved. South-South collaboration, still at an early stage, must be continued.
